# Prediction of a Panel of Programmed Cell Death Protein-1 (PD-1) Inhibitor–Sensitive Biomarkers Using Multiphase Computed Tomography Imaging Textural Features: Retrospective Cohort Analysis

**DOI:** 10.2196/67379

**Published:** 2025-07-11

**Authors:** Shiqi Wang, Na Chai, Jingji Xu, Pengfei Yu, Luguang Huang, Quan Wang, Zhifeng Zhao, Bin Yang, Jiangpeng Wei, Xiangjie Wang, Gang Ji, Minwen Zheng

**Affiliations:** 1 Department of Digestive Surgery, Xijing Hospital of Digestive Diseases, The First Affiliated Hospital of Fourth Military Medical University (Xijing Hospital), Fourth Military Medical University, Xi'an, China; 2 Department of Radiology, The First Affiliated Hospital of Fourth Military Medical University (Xijing Hospital), Fourth Military Medical University, Changlexi St. 127#, Xi'an, 710032, China, 86 2984771533, 86 2984771533; 3 Department of Medical information, The First Affiliated Hospital of Fourth Military Medical University (Xijing Hospital), Fourth Military Medical University, Xi'an, China; 4 Ambulatory Surgery Center, The First Affiliated Hospital of Fourth Military Medical University (Xijing Hospital), Fourth Military Medical University, Xi'an, China

**Keywords:** biomarkers, computed tomography, gastric cancer, immunotherapy, radiomics

## Abstract

**Background:**

Immune checkpoint inhibitors represent an effective therapeutic approach for advanced gastric cancer. Their efficacy largely depends on the status of tumor biomarkers including human epidermal growth factor receptor 2 (HER2), programmed death-ligand 1 (PD-L1; combined positive score ≥1), and microsatellite instability-high (MSI-H). To noninvasively evaluate these biomarkers, researchers have developed radiomic models for individual biomarker prediction. However, in clinical practice, holistic prediction of these biomarkers as an integrated system is more efficient. Currently, the feasibility of implementing radiomics-based comprehensive biomarker prediction remains unclear, requiring further investigation.

**Objective:**

This study aimed to develop a radiomics-based predictive model using multiphase computed tomography (CT) images to holistically evaluate HER2, PD-L1, and MSI-H status in patients with gastric cancer.

**Methods:**

A retrospective analysis was conducted on 461 patients with gastric cancer who underwent radical gastrectomy between 2019 and 2022. Clinical data, contrast-enhanced CT images (arterial phase [AP] and portal venous phase [PP]), and pathological results were collected. Patients were categorized into two groups: (1) the programmed cell death protein-1 inhibitor panel-positive group, comprising patients with HER2 overexpression, PD-L1 positive, or MSI-H status; and (2) the negative group, comprising patients without HER2 amplification, PD-L1 negative, or microsatellite instability-low or microsatellite stable condition. Radiomic features (including first-order statistics, shape features, and wavelet-derived textures) were extracted from both AP and PP images, yielding 1834 features per phase. Least absolute shrinkage and selection operator regression was applied to select key features. In total, 3 models were constructed using the Extreme Gradient Boosting algorithm: AP-only (8 features), PP-only (22 features), and a fused model combining AP and PP features (20 features: 6 AP and 14 PP features). Model performance was evaluated using area under the curve (AUC), sensitivity, specificity, and decision curve analysis.

**Results:**

Of the 461 patients, 147 patients (31.9%) were classified into the panel-positive group. The clinical features were similar between the 2 groups. The fused model demonstrated superior performance in the test set (AUC 0.82, 95% CI 0.68‐0.95), significantly outperforming AP-only (AUC 0.61, 95% CI 0.47‐0.74) and PP-only models (AUC 0.70, 95% CI 0.49‐0.91). Sensitivity and specificity for the AP-only, PP-only, and the fused model were 0.33 and 0.85; 0.50 and 0.86; and 0.60 and 0.83, respectively. Decision curve analysis confirmed that the fused model provided higher clinical net benefit across threshold probabilities.

**Conclusions:**

The construction of integrated biomarker prediction models through radiomics demonstrates technical feasibility, offering a promising methodology for comprehensive tumor characterization.

## Introduction

Gastric cancer remains a leading cause of cancer-related mortality worldwide [1]. Patients with advanced gastric cancer have poor prognoses. Conventional therapies, such as surgery and chemotherapy, show limited effectiveness. The 5-year survival rate for these patients is only 40% [2,3]. Recent advancements in immunotherapy, notably the application of programmed cell death protein-1 (PD-1) inhibitors, have markedly improved patient prognosis. These agents not only effectively suppress tumor progression but also enable surgical intervention in selected cases of late-stage disease.

Many previous studies have shown that programmed death-ligand 1 (PD-L1) positive expression (combined positive score [CPS] ≥1) [4,5], human epidermal growth factor receptor 2 (HER2) overexpression [6-8], and microsatellite instability-high (MSI-H) status all indicate a higher sensitivity to PD-1 inhibitor [9,10]. A meta-analysis revealed that patients with PD-L1-positive expression (CPS≥1) receiving PD-1 inhibitor monotherapy demonstrated significantly improved objective response rates (13% vs 3%) and prolonged overall survival (hazards ratio 0.84) [11]. The KEYNOTE-811 trial further showed that combining PD-1 inhibitor with trastuzumab and chemotherapy in patients with HER2-overexpressing advanced gastric cancer substantially enhanced objective response rates (74.4% vs 51.9%) and progression-free survival (hazards ratio 0.73, 95% CI 0.61‐0.87) [6,7]. In addition, KEYNOTE-059 findings indicated that patients with MSI-H gastric cancer achieved markedly higher objective response rates than non–MSI-H counterparts following PD-1 inhibitor therapy (57.1% vs 9%) [9]. These collective results suggest that PD-1 inhibitors may provide survival benefits in biomarker-positive populations.

For biomarker detection, immunohistochemistry (IHC) remains the gold-standard method, although technical limitations, such as interobserver variability, should be considered. In addition to the low concordance rate between biopsy and resected specimens [12], the invasive nature of biopsy procedures further limits the utility of these tests in patients with coagulopathy, cardiopulmonary dysfunction, or deep-seated tumors. Furthermore, biomarkers exhibit dynamic changes throughout treatment [13], and repeated sampling poses significant challenges for patient compliance.

In recent years, radiomics has enabled noninvasive characterization of tumor biology. Contrast-enhanced computed tomography (CT), a standard imaging modality for gastric cancer staging, captures spatial heterogeneity across entire tumors. These conventional CT images encode critical data about underlying molecular features, including protein expression profiles and genetic alterations [14,15]. Researchers have constructed machine learning models using texture or deep features extracted from preoperative contrast-enhanced CT imaging to predict several biomarkers in patients with gastric cancer: PD-L1 positive expression (area under the curve [AUC] 0.77‐0.78) [16,17], HER2 overexpression (AUC 0.72‐0.91) [18,19], and MSI-H status (AUC 0.76‐0.91) [20-22]. These radiomic approaches demonstrate promising discriminatory performance in noninvasive biomarker assessment.

In clinical practice, candidate selection for PD-1 inhibitor therapy relies on a multimodal evaluation of biomarkers, such as HER2 overexpression, PD-L1 positive expression, or MSI-H status. That is to say, as long as one of the parameters is positive, we can consider using PD-1 inhibitor without verifying each parameter separately. Previous studies have suggested that HER2 overexpression [23,24], MSI-H status [25], and PD-L1 positivity are all related to increased tumor heterogeneity [26], which is reflected in texture features. Accordingly, it will be feasible and more efficient to develop an integrated radiomic model capable of holistic prediction for the aforementioned biomarker panel. In this study, we aim to assess the status of the integrated panel, which includes HER2 overexpression, PD-L1 positive expression, and MSI-H status, using the texture features of preoperative enhanced CT.

## Methods

### Study Design and Participants

Our hospital is a tertiary hospital located in western China. There are 9 surgeons specialized in radical gastrectomy in our department, and over 200 cases of gastrectomy were performed annually. The clinical records of all consecutive patients who underwent radical gastrectomy in our department between January 2019 and July 2022 were retrospectively reviewed. The following clinical features were recorded: sex, age, smoking history, BMI, location of gastric adenocarcinoma, degree of differentiation, and levels of carcinoembryonic antigen.

The inclusion criteria were: advanced stage was verified by the postoperative pathological report (T_3-4_N_0-3_); received IHC test for HER2 overexpression, PD-L1 positive status, and MSI-H status.

The exclusion criteria were: tumor that was invisible in CT images, received any type of antitumor treatment before surgery, and gastric stump cancer. A total of 461 patients were selected for this study.

### Ethical Considerations

This study was approved by the Ethical Committee of the First Affiliated Hospital Of Air Force Military Medical University (20222174-F-1). Dr Yan Jia issued the approval on July 22, 2022. As a retrospective study, the requirement for informed consent was waived. All personal identifiers were removed from the dataset before analysis, and access to raw data was strictly limited to authorized researchers. Confidentiality of participant information was maintained in accordance with the Declaration of Helsinki and relevant data protection regulations throughout the research process.

### HER2, PD-L1, and MSI-H Analysis

The status of HER2, PD-L1, and MSI-H was derived from the postoperative pathology report. These markers were tested by the IHC or additionally by fluorescence in situ hybridization, as appropriate. We defined HER2 overexpression as HER2 IHC 3+ or IHC 2+/fluorescence in situ hybridization–positive, PD-L1 positivity as CPS≥1, and MSI-H status as loss of expression in any of the 4 proteins, that is, MSH2, MSH6, MLH1, and PMS2.

According to the test results of the above parameters, patients were defined as the programmed cell death protein-1 inhibitor panel-positive group (PPP group) when results showed HER2 positive status, CPS≥1, or MSI-H status. Otherwise, patients were classified as the programmed cell death protein-1 inhibitor panel-negative group (PPN group).

### CT Examination

All patients underwent a full abdominal contrast-enhanced CT scan within 3 weeks before surgery. Patients were required to fast for at least 6 hours before the CT examination. Furthermore, 10 minutes before the CT scan, each patient was required to drink 1500 mL of water to distend the stomach. A variety of CT scanners were used to obtain CT images, including 2 from United Imaging (United Imaging Healthcare uCT 760), and 2 from Siemens (Siemens SOMATOM Definition Flash CT). The scanning parameters were as follows: the average acquisition parameter was 110 (ranging from 80 to 140) kVp, exposure time was 751 (ranging from 500 to 1782) milliseconds, and tube current was 170 (ranging from 100 to 450) milliamperes. The thickness of the image reconstruction layers varied from 1 to 5 (averaging 2.5) millimeters. For the enhanced scans, nonionic contrast media (with iodine content of 350 mg/mL and 320 mg/mL, respectively) were used, with an injection rate of 3.5 mL/second, and the total contrast medium volume was 1.2 mL per kilogram of body weight. Arterial phase (AP) and portal venous phase (PP) were scanned at 25 seconds and 50 seconds after injection, respectively.

### Image Acquisition and Standardization

AP and PP contrast-enhanced CT images were retrieved from the institutional Picture Archiving and Communication System (CARESTREAM PACS v8.1.2, Carestream Health Inc) using standardized Digital Imaging and Communications in Medicine export protocols.

To mitigate interscanner heterogeneity across multiple CT platforms, all Digital Imaging and Communications in Medicine datasets were subjected to voxel resampling using a trilinear interpolation algorithm in 3D Slicer (version 5.4.0), an open-source software platform developed and maintained by the Surgical Planning Laboratory at Brigham and Women's Hospital, Harvard Medical School (Boston, MA, USA), with collaborative support from Kitware, Inc. and contributions from a global research consortium. This standardized the spatial resolution to isotropic 1.0 mm³ voxels (x-, y-, or z-axes) and normalized Hounsfield Units values using a B-spline deformation field, ensuring consistency in texture feature extraction across heterogeneous acquisition protocols.

### Tumor Segmentation Protocol

In total, 2 fellowship-trained abdominal radiologists (NC and JX), each with 7 and 12 years of experience in gastrointestinal oncology imaging respectively, conducted independent volumetric tumor delineation under blinded conditions to pathological and molecular data.

On the AP and PP images, the radiologists meticulously traced tumor boundaries on the largest cross-sectional area of the tumor using the 3D Slicer’s Segment Editor module. During the manual delineation process, the selection of the region of interest carefully avoided areas of gastric air, necrosis, and adipose tissue.

One radiologist (NC) manually segmented the tumor region on all CT slices in the largest cross-sectional area using 3D Slicer software (version 5.4.0), after which the segmentation was secondarily reviewed and edited by the second radiologist (JX). The intra- and interobserver reproducibility was assessed by calculating the interclass and intraclass correlation coefficients (ICCs) of the first-order texture features (energy, entropy) and Gray-Level Co-occurrence Matrix features.

### Texture Feature Extraction and Analysis

We used the PyRadiomics package (version 3.7.12) and the scikit-learn (version 0.22) package to systematically extract radiomic features from the manually segmented regions of interest. The extraction process was performed on both the AP and PP phases of the contrast-enhanced CT images. The radiomic features included a comprehensive set of histogram-based features (eg, mean, SD, skewness, kurtosis, and percentiles) and wavelet texture features (eg, energy, entropy, and correlation), capturing both intensity distribution and spatial heterogeneity of the tumor.

To ensure robustness and reproducibility, the feature extraction pipeline was implemented with the following standardized settings. First, Gray-Level Discretization: the raw pixel intensity values were discretized into 256 bins using a uniform quantization method to reduce noise and enhance feature stability. Second, wavelet decomposition: features were extracted at 3 decomposition levels (low pass - low pass [characterizes smooth approximation], low pass - high pass [characterizes vertical details], high pass - low pass [characterizes horizontal details], high pass - high pass [characterizes diagonal details]) using the Haar wavelet transform to capture multiscale texture information. Third, neighborhood settings: a 3D isotropic kernel with a radius of 1 voxel was applied to compute neighborhood-based texture features, ensuring consistency across different tumor sizes. Through the aforementioned extraction process, we obtained AP features and PP features. Furthermore, we achieved dual-phase feature fusion by means of horizontal concatenation of feature vectors, thereby acquiring integrated fusion features.

The feature selection process was conducted in a multistep, hierarchical manner to identify the most stable, discriminative, and nonredundant features: (1) reproducibility assessment: intra- and interreader reproducibility was evaluated on a resegmentation dataset comprising 30 randomly selected cases. The intraclass correlation coefficient (ICC) was calculated for each feature, with features demonstrating ICC >0.75 for both intra- and interreader reproducibility retained for subsequent analysis. (2) All radiomic features were normalized using *z* score standardization to ensure comparability across different imaging protocols and scanners. (3) Statistical screening: a 2-tailed independent samples *t* test was performed to compare the feature distributions between the PD-1 inhibitor PPP and PPN groups. Features with a *P* value<.05 were selected as potentially discriminative. (4) Redundancy removal: a correlation analysis was conducted to eliminate redundant features with high collinearity. A Pearson correlation coefficient threshold of 0.9 was applied, retaining only 1 feature from each highly correlated pair.

### Feature Sparsity and Penalized Regression

First, the least absolute shrinkage and selection operator (LASSO) logistic regression algorithm was applied to identify the most predictive features while controlling for model complexity. Second, the penalty parameter (λ) was optimized using a 10-fold cross-validation procedure, with λ tuned to minimize the cross-validation error. Third, the optimal λ value was determined based on the “one-standard-error” rule, ensuring a balance between model performance and feature parsimony. Finally, features with nonzero coefficients in the LASSO model were selected for final model construction.

### Prediction Model Building

The feature selection and modeling processes were conducted in the training set, with feature selection performed using LASSO regression to iteratively screen and optimize penalty coefficients, thereby reducing dimensionality and extracting important features.

The Extreme Gradient Boosting (XGBoost) algorithm, a gradient-boosted decision tree-based method, was used to develop the classification models, with hyperparameters such as learning rate, tree depth, and subsample ratio optimized through grid search or random search to enhance model performance. In addition, several other algorithms, such as random forest, linear regression, and support vector machines, were also used.

Model performance was comprehensively evaluated using the area under the receiver operating characteristic curve, AUC, sensitivity, and specificity. To demonstrate clinical utility, decision curve analysis was performed to evaluate net benefit across different threshold probabilities and to assess clinical net benefit. The efficacy of the constructed model was evaluated using 10-fold cross-validation with stratified sampling. In addition, receiver operating characteristic curves and calibration curves were plotted to further assess model discrimination and calibration, ensuring reliable and accurate results.

### Statistical Analysis

Categorical data were reported as numbers with proportions, and quantitative data were reported as the mean with SD. Categorical data were compared using the chi-square test or Fisher exact test, where appropriate. For continuous data, the Student *t* test or Mann‒Whitney *U* test was used. A 2-sided *P* value of <.05 was considered statistically significant. AUC was used to assess the performance of each model.

## Results

### Clinical Characteristics

The study population consisted of 461 patients who underwent radical gastrectomy. The proportion of patients with HER2 overexpression, PD-L1 positive status (CPS≥1), and MSI-H status was 5.4% (25/461), 24.1% (111/461), and 5.6% (26/461), respectively. Based on HER2, PD-L1, and MSI-H status, 32.2% (147/461) of patients were classified into the PPP group. The demographic data and clinical characteristics of the patients are listed in Table 1.

**Table 1.** Clinical features of included patients.

	PPP^a^ group (n=149)	PPN^b^ group (n=312)	*P* value
Sex (male), n (%)	114 (76.5)	237 (76)	.90
Age (years), mean (SD)	59.93 (10.74)	59.26 (10.74)	.54
BMI (kg/m^2^), mean (SD)	23.65 (3.21)	23.71 (3.52)	.87
Smoking, n (%)	49 (32.9)	115 (36.9)	.41
GEJ^c^ cancer, n (%)	49 (32.9)	112 (35.9)	.53
Poorly differentiated carcinoma, n (%)	112 (75.2)	237 (76)	.85
Mucinous, n (%)	11 (7.4)	30 (9.6)	.43
CEA^d^ (ng/mL), mean (SD)	10.83 (38.05)	9.51 (30.61)	.69
NEUT#^e^ (10E9/L), mean (SD)	7.14 (5.05)	6.86 (4.18)	.56
LYM#^f^ (10E9/L), mean (SD)	1.22 (0.61)	1.14 (0.59)	.16
Tumor (T3^g^), n (%)	82 (55)	138 (44.2)	.04
N^h^ (+), n (%)	111 (74.5)	253 (81.1)	.10

a PPP: programmed cell death protein-1 inhibitor panel-positive.

b PPN: programmed cell death protein-1 inhibitor panel-negative.

c GEJ: gastroesophageal junction.

d CEA: carcinoembryonic antigen.

e NEUT#: neutrophil absolute value.

f LYM#: lymphocyte absolute value.

g T3 indicates that the tumor has penetrated the muscularis propria of the gastric wall with potential invasion into the subserosal tissue or adjacent adipose tissue, but without breaching the overlying serosal layer.

h N: regional lymph nodes metastasis.

### Retrieved CT Texture Features and Feature Selection

**Figure 1.**
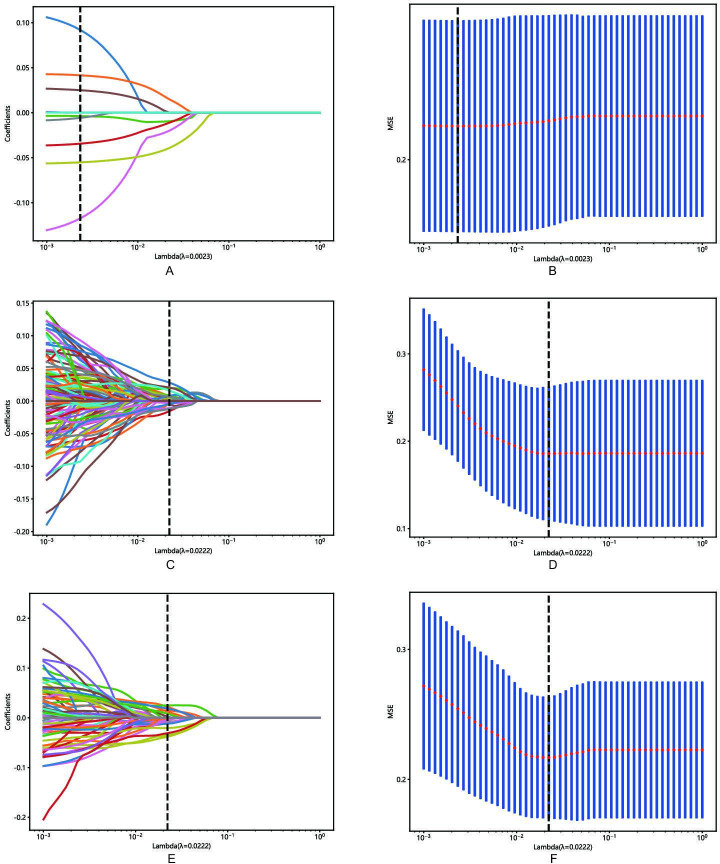
Radiomics feature selection using the least absolute shrinkage and selection operator regression model. (**A**) Least absolute shrinkage and selection operator method was used to confirm the optimal adjustment parameter λ for arterial phase, (**C**) portal venous phase, and (**E**) fusion features. (**B**) Least absolute shrinkage and selection operator coefficient profiles of the selected radiomics features of arterial phase, (**D**) portal venous phase, and (**F**) fusion features. The vertical line was drawn according to the value selected by 10-fold cross-validation. MSE: mean squared error.

**Figure 2.**
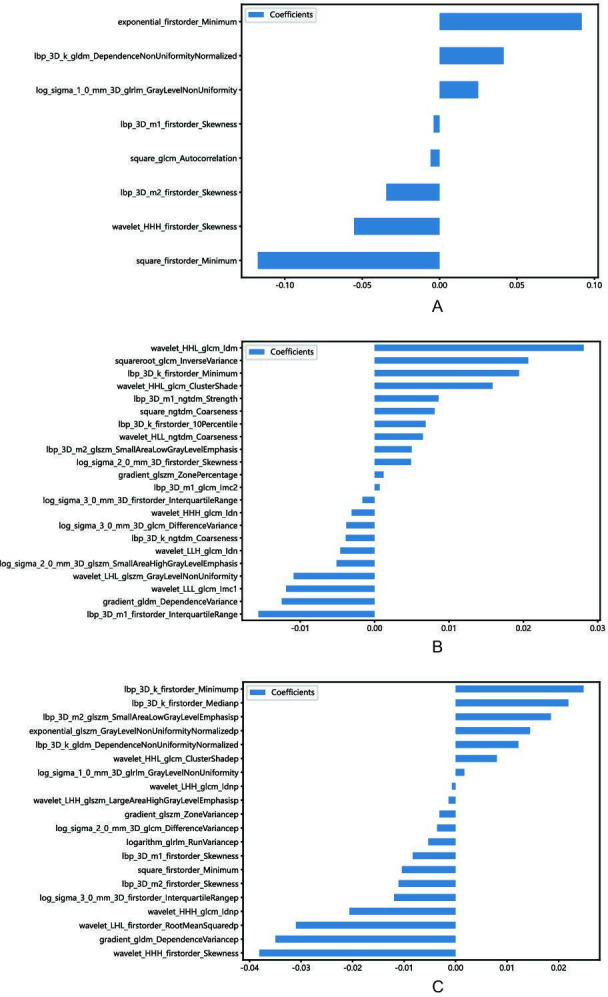
The chosen subset of radiomics features. The 8 of the arterial phase features, 22 of the portal venous phase features, and 20 of the fusion features with nonzero coefficients were chosen, and the corresponding coefficients were evaluated.

A total of 1834 features were retrieved from the segmentation areas on the AP or PP CT images, respectively. The features included first-order features, shape features, and wavelet texture features. Reproducibility analysis retained 1325 and 1506 features with an ICC >0.75 from AP and PP CT images, respectively. The AP and PP features were horizontally concatenated to construct the fusion dataset, and *z* score standardization was performed for each dataset. Following a 2-tailed independent samples *t* test, 11, 666, and 210 features from the AP, PP, and fusion datasets were retained, respectively (Multimedia Appendix 1). Correlation analysis to remove redundant features further reduced these to 10, 147, and 89 features for the AP, PP, and fusion datasets, respectively. Subsequently, LASSO regression was applied to select the most relevant features. The LASSO path plot for the training set is presented in Figure 1, with the relative weights of the selected features shown in Figure 2. Ultimately, feature selection yielded 8 AP features, 22 PP features, and 20 fusion features (comprising 6 AP and 14 PP features).

### Model Construction and Evaluation in Assessing the ICC Panel

**Figure 3.**
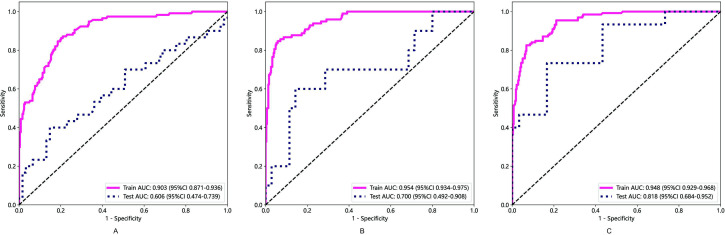
The performance of different models in identifying the PD-1 inhibitor panel-positive group. (**A**) Receiver operating characteristic curves of arterial phase features derived model in the training set and testing set; (B) Receiver operating characteristic curves of portal venous phase features derived model in the training set and testing set; (C) Receiver operating characteristic curves of fusion features derived model in the training set and testing set. AUC: area under the curve; PD-1: programmed cell death protein-1.

The selected features were used to construct classification models. Table 2 lists the classification performances of the AP, PP, and fusion features based models (XGBoost algorithms) both in the training and the testing sets. In the testing set, the AP features-based model predicted the PPP group with AUC of 0.61 (95% CI 0.47‐0.74), sensitivity of 0.33, and specificity of 0.85; the PP model showed AUC of 0.7 (95% CI 0.49‐0.91), sensitivity of 0.50, and specificity of 0.86; the fusion model performed better, with an AUC of 0.82, (95% CI 0.68‐0.95), sensitivity of 0.60, and specificity of 0.83. The performances of the models are illustrated in Figure 3. The performances of other models are demonstrated in Multimedia Appendix 2.

**Table 2.** Predictive performances of the radiomics models in the training and testing set.

	AUC^a^ (95% CI)	Accuracy	Sensitivity	Specificity
AP^b^ model^c^	0.90 (0.87‐0.94)	0.81	0.86	0.79
AP model	0.61 (0.47‐0.74)	0.68	0.33	0.85
PP^d^ model^c^	0.95 (0.93‐0.98)	0.92	0.84	0.94
PP model	0.70 (0.49‐0.91)	0.78	0.50	0.86
Fusion model^c^	0.95 (0.93‐0.97)	0.90	0.82	0.93
Fusion model	0.82 (0.68‐0.95)	0.76	0.60	0.83

a AUC: area under the curve.

b AP: arterial phase.

c Training set.

d PP: portal venous phase.

**Figure 4.**
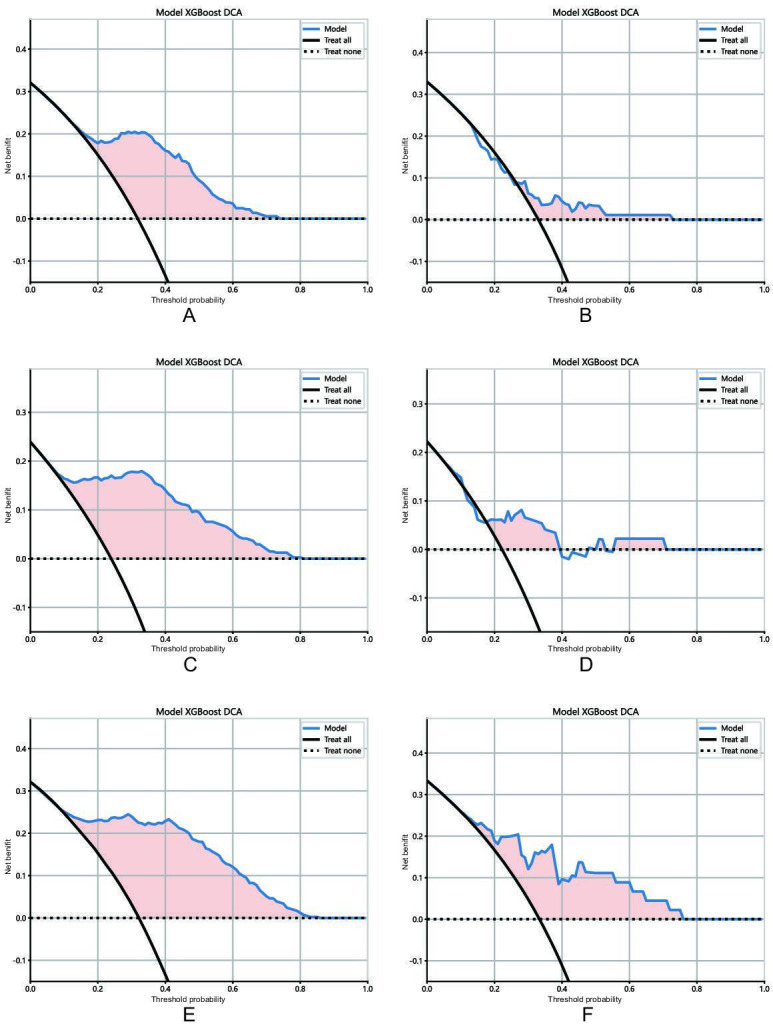
DCA of the derived models in the training set and testing set. (**A**) Arterial phase features derived model in the training set and (**B**) testing set; (**C**) portal venous phase features derived model in the training set and (**D**) testing set; (**E**) fusion features derived model in the training set and (**F**) testing set. DCA: decision curve analysis; XGBoost: Extreme Gradient Boosting.

The decision curves demonstrated that the fusion model may derive more net benefits to patients than the sole AP or PP features based models (Figure 4).

## Discussion

### Principal Results

Based on texture features from multiphase CT images, this study first develops a radiomics model that holistically evaluates HER2, PD-L1, and MSI-H status in patients with gastric cancer. By using texture features from both the AP and PP images, the holistic prediction model (XGBoost algorithms) demonstrated an AUC of 0.95 in the training set and 0.82 in the testing set. These results demonstrate the feasibility of radiomics models in achieving holistic prediction for a panel of biomarkers, thereby enhancing the assessment efficiency.

### Limitations

There are some limitations in our study. First, this is a single-center study with a relatively small sample size. Second, the predictive performance of the model was evaluated solely through internal cross-validation rather than external validation, introducing potential overfitting risks. Owing to the retrospective design and limited sample size, we currently lack access to independent external datasets for validation. Therefore, future studies should rigorously assess the model’s predictive performance by incorporating external validation and calibration curve analysis. Third, 2D segmentation on a single slice was applied instead of 3D segmentation encompassing the entire tumor. However, this less labor-intensive approach is recommended in radiomic studies of gastric cancer [27].

### Comparison With Previous Work

Several studies have proved the efficacy of enhanced CT image texture features in predicting PD-L1 expression (AUC 0.74-0.77) [16,17], HER2 overexpression (AUC 0.79-0.83) [18,19], or MSI-H status (AUC 0.76-0.91) [20-22]. Our study demonstrated comparable AUC (0.82) in predicting the biomarker as an integrated panel. These findings demonstrate that the integrated model maintains robust predictive performance while improving assessment efficiency.

The primary distinction from previous research lies in our comprehensive evaluation of biomarkers as an integrated panel. Although aggregating patients with diverse biomarker profiles as a single cohort may obscure heterogeneity in treatment sensitivity to PD-1 inhibitor among individuals within the same group, this does not preclude sensitivity evaluation or clinical application of PD-1 inhibitor therapy in this group. Furthermore, collective analysis of these biomarkers could potentially enhance the predictive capacity of radiomics. First, it improves the clinical applicability. As long as one of the parameters is positive, we can consider using the PD-1 inhibitor without verifying each parameter separately. Second, it enhances the stability of the model. Due to the limited rates of HER2 overexpression and MSI-H status, previous studies often encountered imbalanced outcome events, and too few positive events may weaken the robustness of the model. For example, patients with HER2 amplification constituted only 19.2% (198/1033) of the population [28]. Patients with MSI-H status accounted for only 9% (37/396) of the overall cohort [20]. Although methods, such as synthetic minority oversampling technique, can be used to handle imbalanced datasets before constructing the model, a better approach is to ensure an adequate number of positive events. In our study, the positivity rate of the ICC panel reached 32.2%, which may enhance the robustness of the model in this aspect. Third, considering these parameters as a panel may theoretically improve the predictive performance of the model. The fundamental rationale for employing radiomics in biomarker classification may lie in distinguishing heterogeneity levels. Since factors such as HER2 overexpression, PD-L1 positivity, or MSI-H may elevate tumor heterogeneity [21,29,30], independent grouping based on any single biomarker could increase heterogeneity within control cohorts, thereby compromising the model’s discriminative capacity. Classifying these biomarkers as an integrated entity may mitigate confounding effects arising from suboptimal heterogeneity discrimination during the categorization process.

Previous models showed that increased tumor heterogeneity can be reflected by some texture features, such as log.sigma.2.5.mm.3D glrlm run entropy, wavelet.LLL Gray-Level Co-occurrence Matrix difference entropy [17], log.sigma.3.0.mm.3D GLSZM zone entropy [16], GLSZM gray level non uniformity, and size zone non uniformity [20,21]. This study demonstrated similar results. We found that the 3 features that most strongly correlated with the integrated model were wavelet HHH first order skewness (AP), gradient gldm dependence variance (PP), and wavelet LHL first order root mean square (PP), and these features characterize tumor spatial and metabolic heterogeneity by quantifying the symmetry of regional gray-level distribution, the dispersion degree of adjacent pixel intensity variations, and the overall energy intensity of regional signals, respectively.

CT images acquired at different contrast phases may yield complementary radiomic parameters. To leverage this advantage, features extracted from both AP and PP were integrated into the analysis. Previous studies predominantly extracted features from either venous phase [16,17,20], AP [18], or PP images individually [22], while others combined features across multiple phases [19,21]. However, no comparative analysis has been conducted to determine the optimal imaging phase. To identify the most suitable phase for biomarker assessment, we developed separate models using AP and PP features, along with an early-fusion model incorporating both phases. Our results demonstrate that PP images provide more discriminative texture features for biomarker evaluation, yielding superior predictive performance compared with AP models. Furthermore, feature fusion from both phases significantly enriched the texture feature repertoire for model construction and enhanced predictive efficacy (Table 2). The underlying reason may lie in the fact that AP and PP images capture distinct tumor characteristics, thereby achieving complementary information gain. For instance, AP features emphasize vascular-related texture patterns (eg, microvascular density variations), while PP features predominantly reflect morphological attributes and stromal interactions (eg, extracellular matrix distribution). Integrating dual-phase signatures enhances model robustness by mitigating overfitting risks inherent in single-phase feature extraction. These findings underscore the clinical value of multiphase feature integration.

Regarding algorithm selection, multiple methods (including random forest [16-19], support vector machines [18], and linear regression [20-22]) have been employed for classification model development. However, no consensus exists regarding the optimal algorithm for classification performance. Our systematic comparison of multiple machine learning approaches revealed that XGBoost-based models demonstrated superior classification accuracy (Multimedia Appendix 2). This advantage likely stems from three technical innovations within XGBoost’s gradient boosting framework: (1) regularization robustness: integration of L1/L2 regularization with randomized subsampling (row and column subsampling) effectively controls model complexity, ensuring stable performance with heterogeneous medical data containing missing values and outliers; (2) computational efficiency: parallelized feature splitting and sparse data-aware architecture enable efficient processing of high-dimensional biomarkers without manual feature selection; and (3) clinical data adaptability: native handling of missing values mitigates imputation bias, while the scale_pos_weight parameter directly addresses class imbalance in disease cohorts. However, algorithm selection should be task-specific, as no universally optimal solution exists across all clinical prediction scenarios.

To enhance predictive performance, some studies have incorporated clinical characteristics and radiomic features [16,18,20,22]. However, the integration of clinical variables does not universally improve model efficacy. For instance, 1 MSI-H prediction model demonstrated no AUC improvement after adding clinical parameters (from 0.91 to 0.89) [22]. In our study, although the PPP group exhibited a higher proportion of T3 staging, we were unable to accurately distinguish between T3 and T4 stages based on CT imaging characteristics. Furthermore, no statistically significant differences were observed in other clinical parameters between the 2 groups. Consequently, clinical features were not incorporated into the predictive model.

### Conclusions

This study demonstrates the feasibility of a multiphasic CT-based radiomics model for the holistic prediction of HER2, PD-L1, and MSI-H status in patients with gastric cancer. The proposed model exhibits predictive performance comparable with conventional single-biomarker approaches while demonstrating superior operational efficiency. Our analysis incorporated both AP and PP CT imaging features, with their combined integration yielding significantly enhanced predictive accuracy relative to single-phase methodologies. However, comprehensive clinical validation remains necessary to establish the model’s utility for evaluating PD-1 inhibitor therapeutic efficacy in real-world settings.

## Supplementary material

10.2196/67379 Multimedia Appendix 1 (A) T-test for the features extracted from the arterial phase images; (B) T-test for the features extracted from the portal venous phase images; (C) T-test for the features extracted from the arterial and portal venous phase images.

10.2196/67379 Multimedia Appendix 2 (A) Performance of arterial phase features based models in the training set; (B) performance of arterial phase features based models in the testing set; (C) performance of portal venous phase features based models in the training set; (D) performance of portal venous phase features based models in the testing set; (E) performance of fused features based models in the training set; (F) performance of fused features based models in the testing set.

## Abbreviations

AP: arterial phase
AUC: area under the curve
CPS: combined positive score
CT: computed tomography
HER2: human epidermal growth factor receptor 2
ICC: intraclass correlation coefficient
IHC: immunohistochemistry
LASSO: least absolute shrinkage and selection operator
MSI-H: microsatellite instability-high
PD-1: programmed cell death protein-1
PD-L1: programmed death-ligand 1
PP: portal venous phase
PPN: programmed cell death protein-1 inhibitor panel negative
PPP: programmed cell death protein-1 inhibitor panel positive
XGBoost: Extreme Gradient Boosting
