# Supplementary material for: Prediction of a Panel of Programmed Cell Death Protein-1 (PD-1) Inhibitor–Sensitive Biomarkers Using Multiphase Computed Tomography Imaging Textural Features: Retrospective Cohort Analysis
Source: JMIR Cancer. 2025 Jul 11;11:e67379. doi: 10.2196/67379 (PMC12274051; doi:10.2196/67379)
Supplement: Multimedia Appendix 1 [file cancer-v11-e67379-s001.pdf]

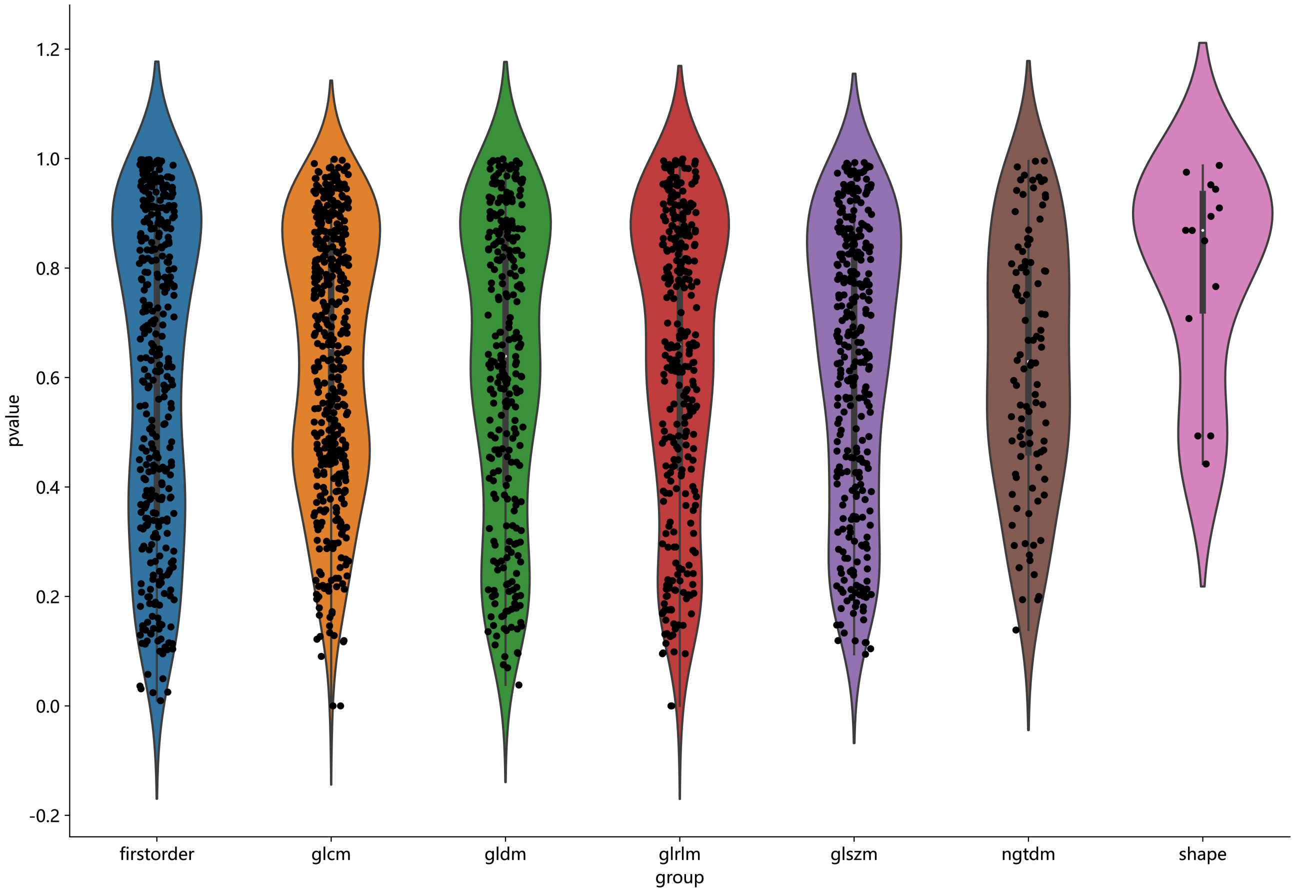

A. T-test for the features extracted from the arterial phase images.

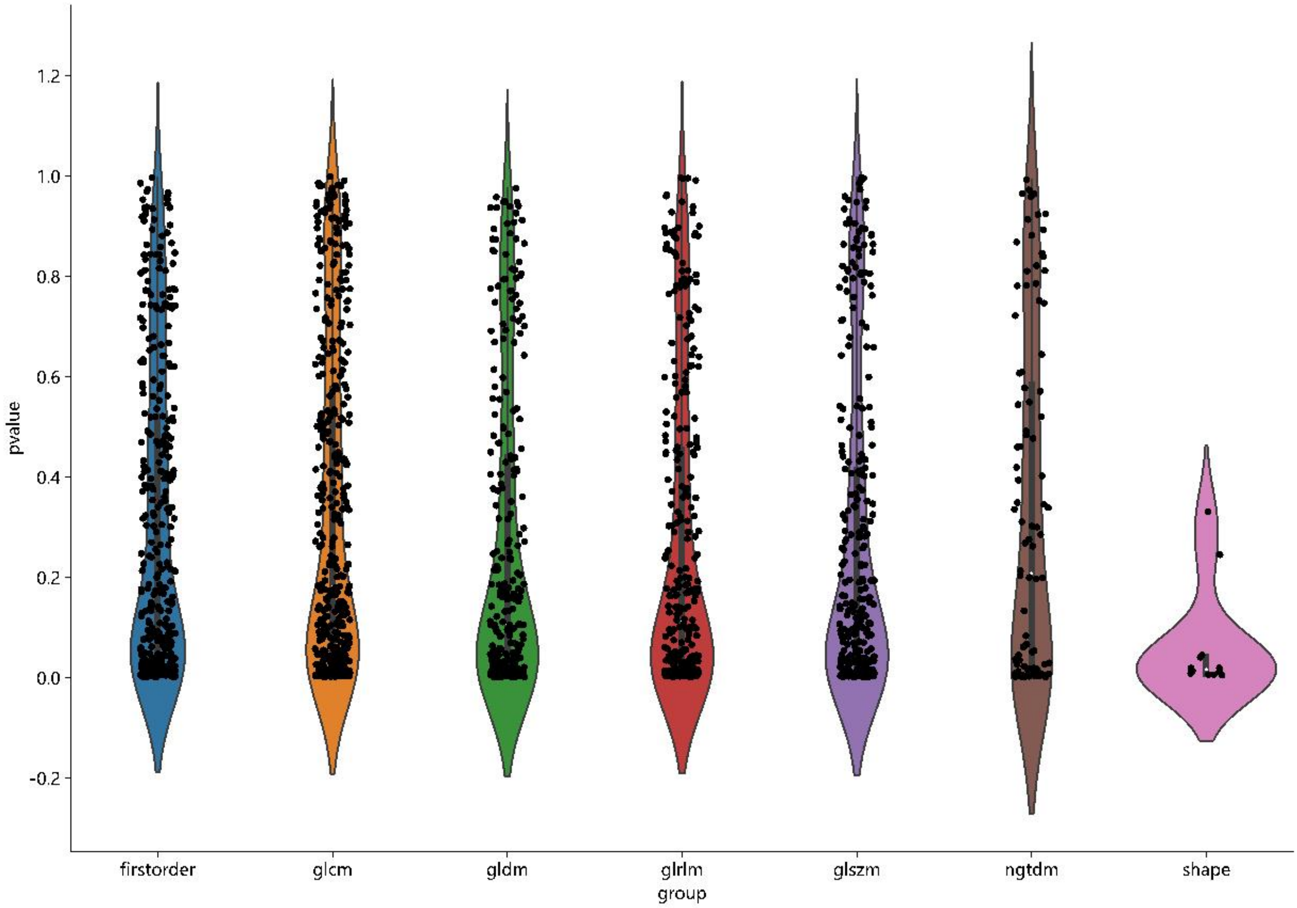

B. T-test for the features extracted from the portal vein phase images

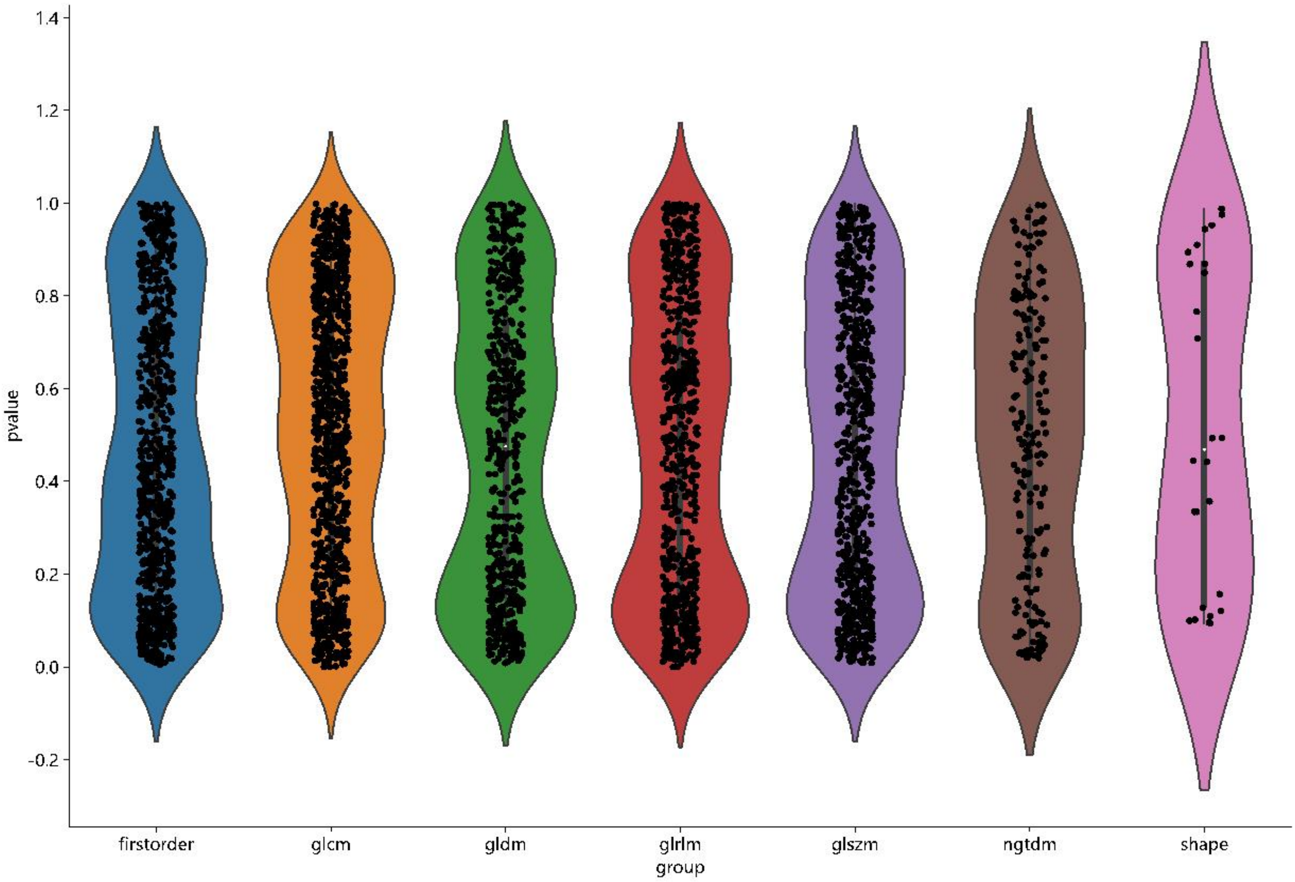

C. T-test for the features extracted from the arterial and portal vein phase images.
