# Supplementary figures and images for: Prediction of a Panel of Programmed Cell Death Protein-1 (PD-1) Inhibitor–Sensitive Biomarkers Using Multiphase Computed Tomography Imaging Textural Features: Retrospective Cohort Analysis
Source: JMIR Cancer. 2025 Jul 11;11:e67379. doi: 10.2196/67379 (PMC12274051; doi:10.2196/67379)

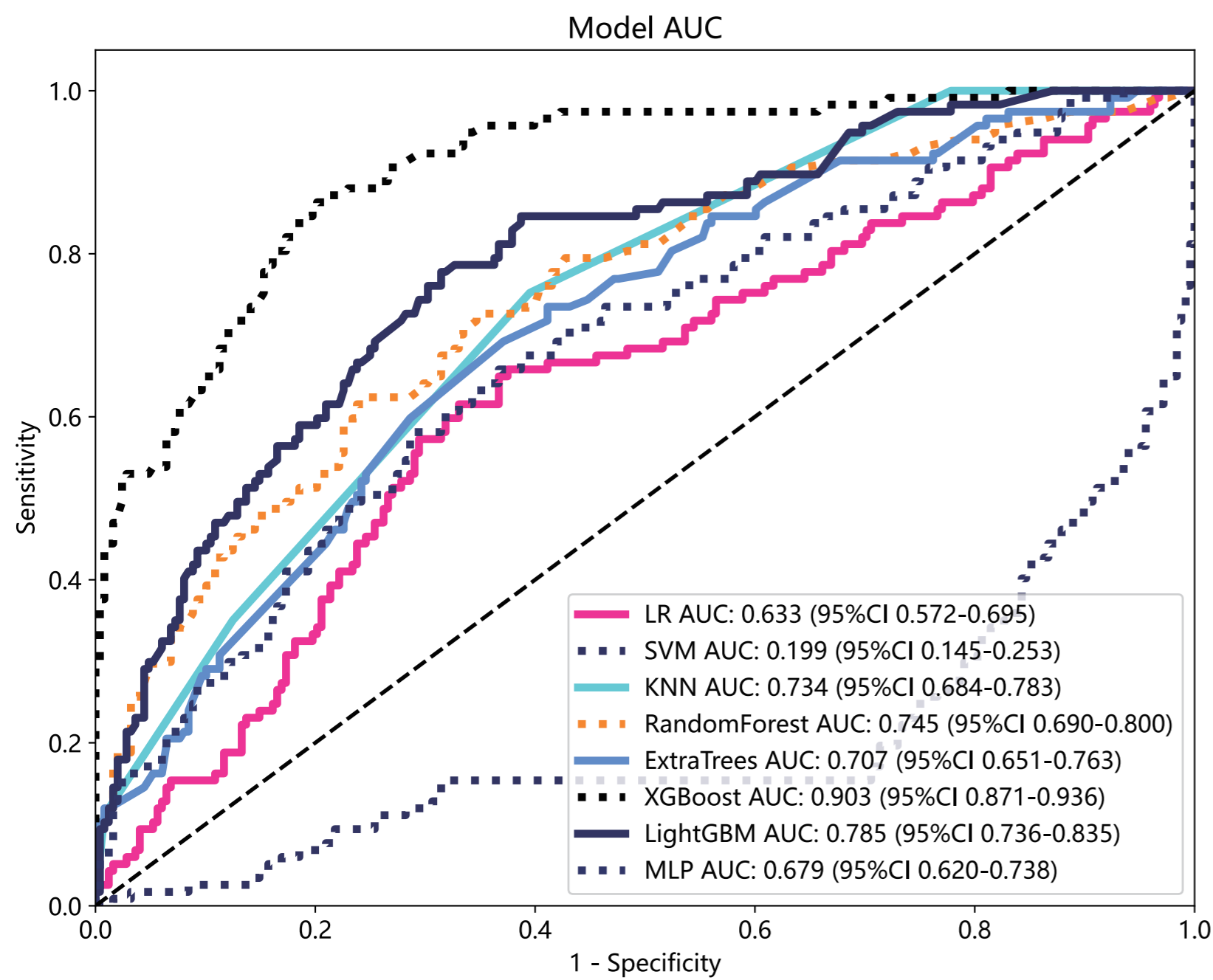

A

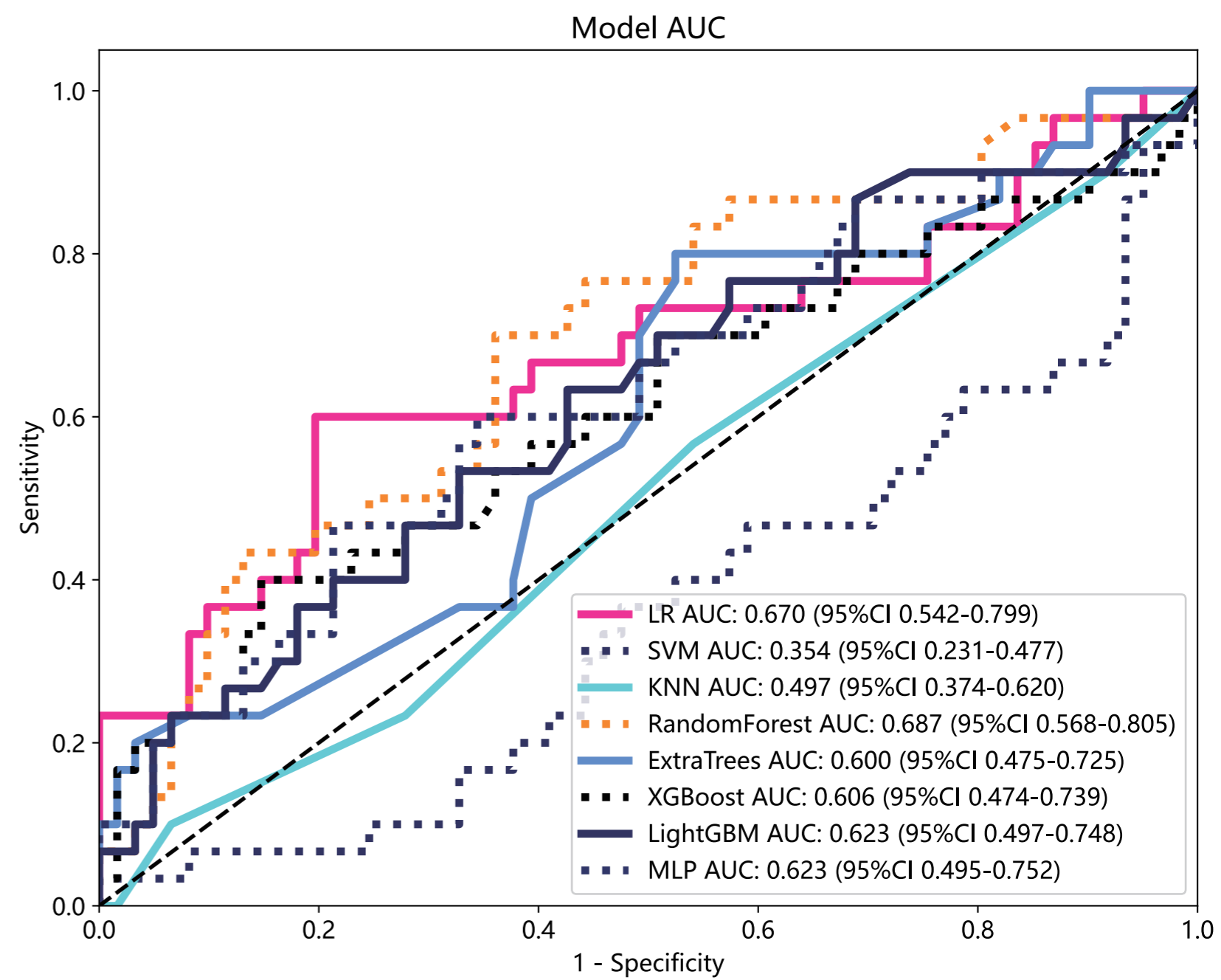

B

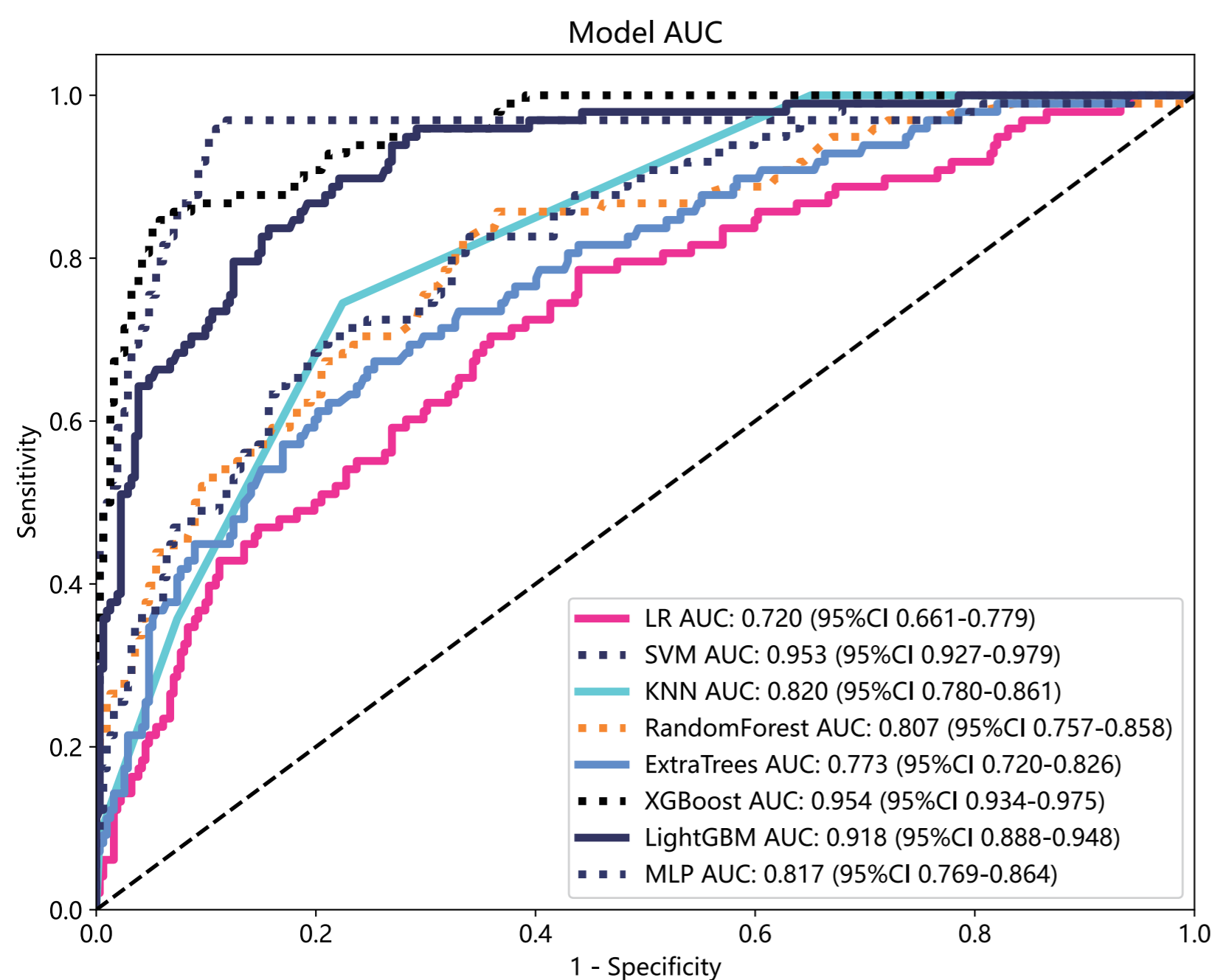

C

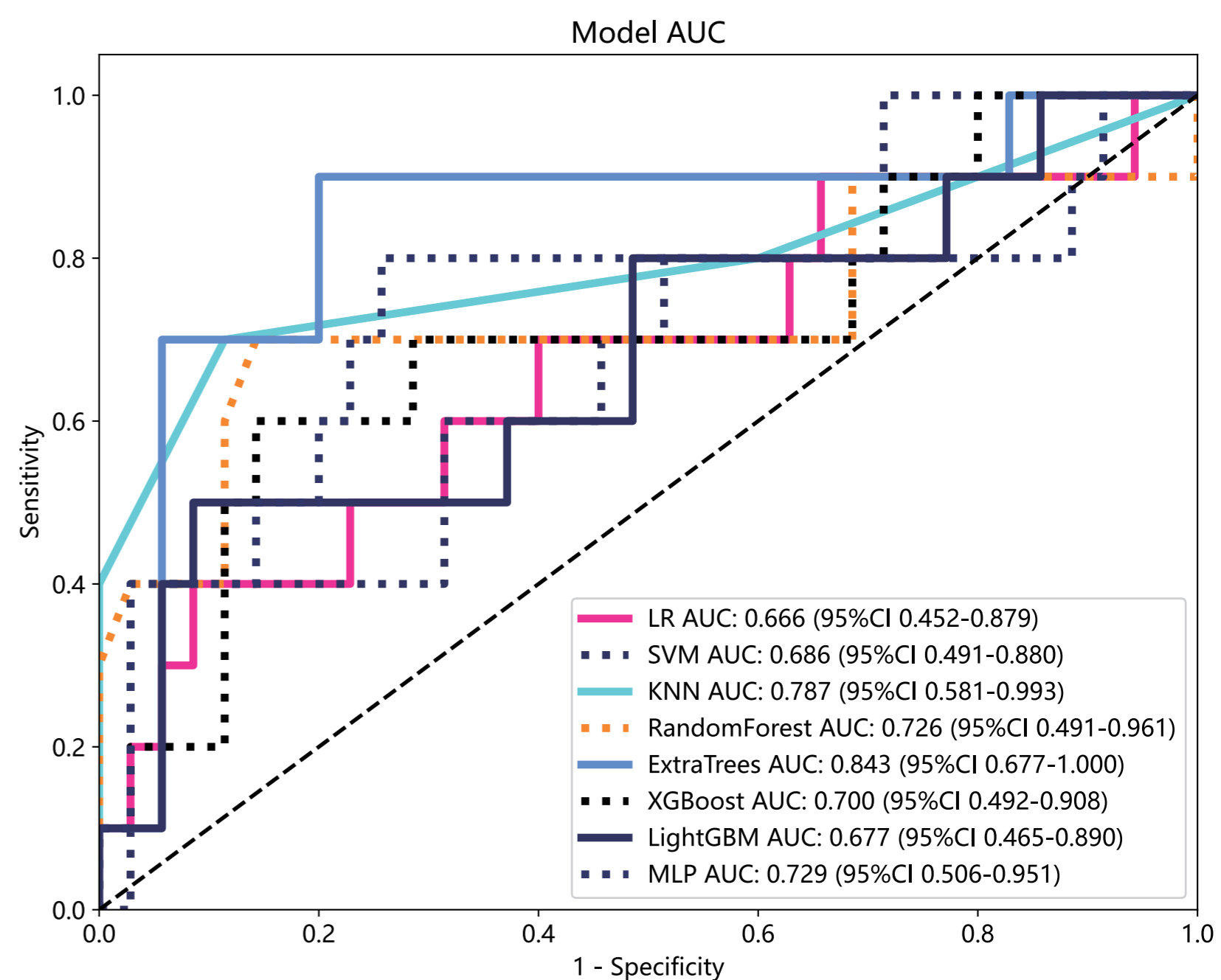

D

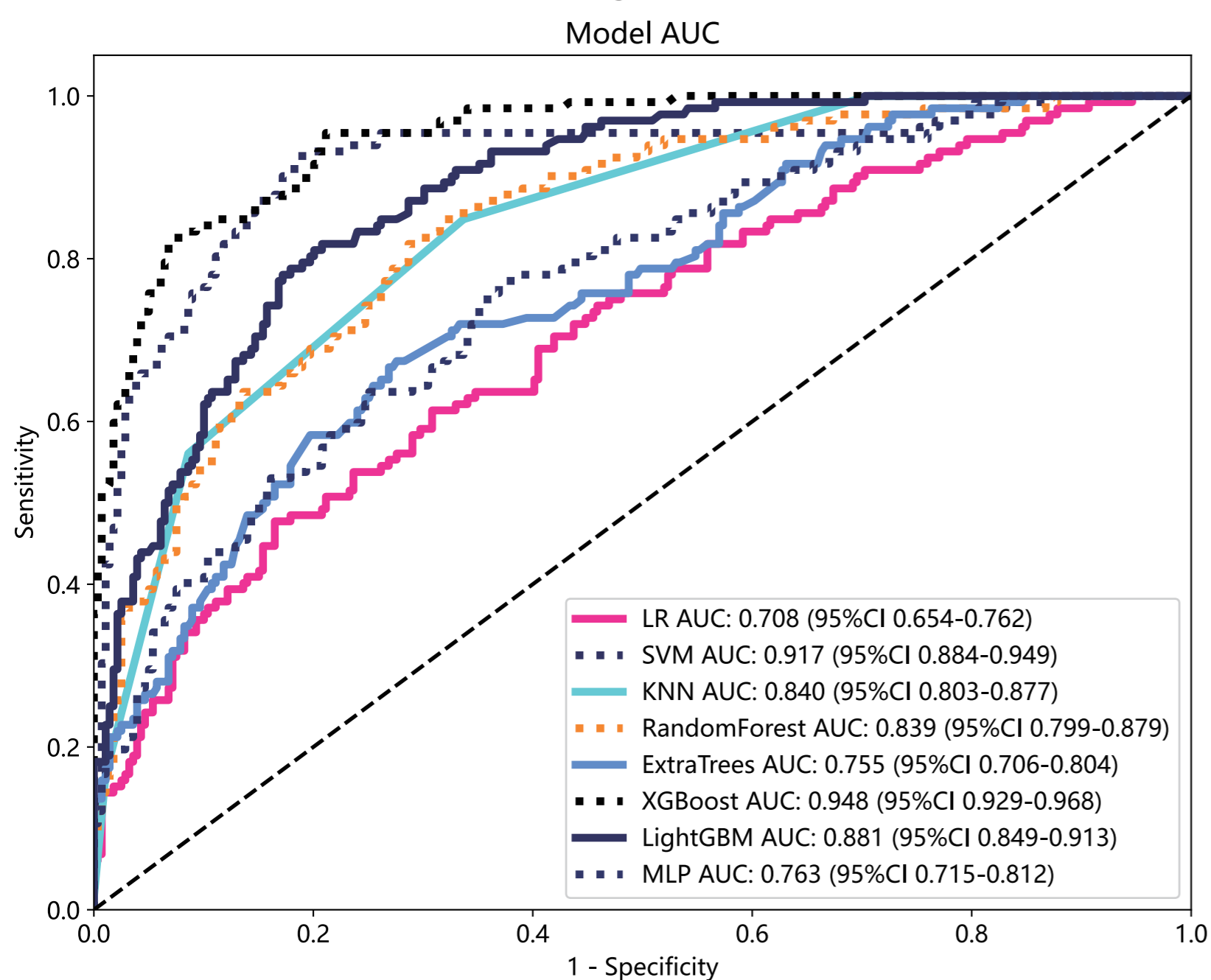

E

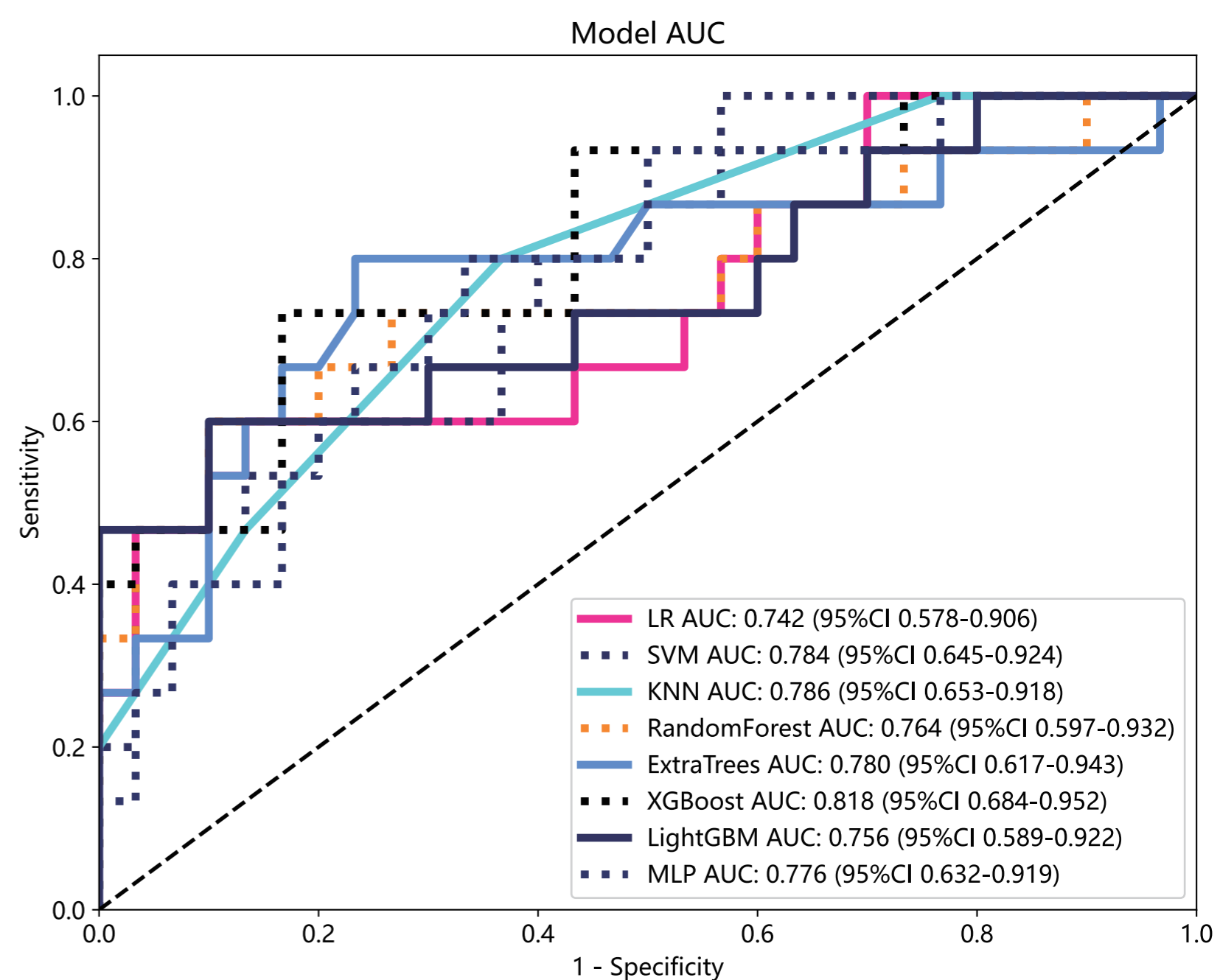

F

Supplement: Multimedia Appendix 2 [file cancer-v11-e67379-s002.pdf]
